# Coordination of KSHV Latent and Lytic Gene Control by CTCF-Cohesin Mediated Chromosome Conformation

**DOI:** 10.1371/journal.ppat.1002140

**Published:** 2011-08-18

**Authors:** Hyojeung Kang, Andreas Wiedmer, Yan Yuan, Erle Robertson, Paul M. Lieberman

**Affiliations:** 1 The Wistar Institute, Philadelphia, Pennsylvania, United States of America; 2 The Kyungpook National University, College of Pharmacy, Daegu, Korea; 3 The University of Pennsylvania, School of Dentistry, Philadelphia, Pennsylvania, United States of America; 4 The University of Pennsylvania, School of Medicine, Philadelphia, Pennsylvania, United States of America; Emory University, United States of America

## Abstract

Herpesvirus persistence requires a dynamic balance between latent and lytic cycle gene expression, but how this balance is maintained remains enigmatic. We have previously shown that the Kaposi's Sarcoma-Associated Herpesvirus (KSHV) major latency transcripts encoding LANA, vCyclin, vFLIP, v-miRNAs, and Kaposin are regulated, in part, by a chromatin organizing element that binds CTCF and cohesins. Using viral genome-wide chromatin conformation capture (3C) methods, we now show that KSHV latency control region is physically linked to the promoter regulatory region for ORF50, which encodes the KSHV immediate early protein RTA. Other linkages were also observed, including an interaction between the 5′ and 3′ end of the latency transcription cluster. Mutation of the CTCF-cohesin binding site reduced or eliminated the chromatin conformation linkages, and deregulated viral transcription and genome copy number control. siRNA depletion of CTCF or cohesin subunits also disrupted chromosomal linkages and deregulated viral latent and lytic gene transcription. Furthermore, the linkage between the latent and lytic control region was subject to cell cycle fluctuation and disrupted during lytic cycle reactivation, suggesting that these interactions are dynamic and regulatory. Our findings indicate that KSHV genomes are organized into chromatin loops mediated by CTCF and cohesin interactions, and that these inter-chromosomal linkages coordinate latent and lytic gene control.

## Author Summary

Multiple mechanisms have been implicated in the control of herpesvirus latent and lytic gene regulation, but few mechanisms account for coordinate regulation of these two life cycles. Here, we show that the transcription control elements for KSHV latent and lytic genes are in close physical proximity. Mutations in the CTCF binding sites of the KSHV latency control region caused a loss of cohesin binding, and derepression of latent transcripts. Loss of CTCF binding also caused a loss of KSHV DNA copy number, and a failure to express lytic genes, including the immediate early gene Rta. Chromatin conformation capture (3C) methods indicated that the CTCF binding sites in the latency control region are linked to the promoter region of Rta. Additional chromatin linkages were detected between the 5′ and 3′ ends of the major latency transcripts, suggesting that chromatin loops organize both latent and lytic gene clusters. The interaction between latent and lytic control regions was subject to cell cycle regulation, consistent with earlier studies implicating cell cycle control of cohesin binding and viral transcription patterns. KSHV chromosome conformation was also disrupted by lytic cycle reactivation. We propose that CTCF-cohesin form dynamic linkages between viral regulatory domains to both insulate and coordinate latent and lytic gene expression.

## Introduction

Kaposis's Sarcoma-Associated Herpesvirus (KSHV) is the human gammaherpesvirus identified as the causative agent of Kaposi's Sarcoma (KS), pleural effusion lymphoma (PEL), and Castleman's Disease ([1], [2], [3] (reviewed in [4], [5], [6]). KSHV pathogenesis depends on the long-term persistence of the viral genome and a restricted pattern of viral gene expression characteristic of latent infection. Most forms of latent infection and tissue biopsies express a complex multicistronic transcript encoding the viral genes for LANA (ORF73), vCyclin (ORF72), vFLIP (ORF71), v-miRNAs, and Kaposin (K12) [7], [8]. These genes are required for viral episome maintenance, host-cell survival, and suppression of lytic gene activation [4], [5], [9]. In contrast, viral lytic genes are required for virion production, which is highly restricted in most culture models of gammaherpesviruses. Nevertheless, most KSHV associated tumors, and many cell culture models display a consistent subpopulation of cells that express some lytic genes [10], [11]. The extent to which lytic gene expression is required for maintaining a stable copy number of viral genomes in a population of cells, and the mechanisms that regulate spontaneous lytic gene expression in latently infected cell pools remains enigmatic, yet central to our understanding KSHV persistence and pathogenesis.

The KSHV latent episome is known to be chromatinized and subject to domain structures that may compartmentalize active from inactive regions of the viral genome [12], [13]. We and others have shown that histone modification patterns contribute to the regulation of KSHV latent and lytic gene control [12], [13], [14]. We have found that the vertebrate chromatin insulator protein CTCF binds to a region within the major latency transcript [15]. Deletion of the entire CTCF region led to a loss of KSHV episome stability and deregulation of latency transcription, including aberrant expression of ORF74/vGPCR, a lytic gene implicated in KS pathogenesis. The CTCF binding site was also found to be co-occupied with the three subunits of the cellular cohesin complex, SMC1, SMC3, and Rad21. Further studies showed that cohesin binding was cell cycle regulated and was partly responsible for the cell cycle control of LANA gene expression [16]. While CTCF and cohesin binding to the latency control region is important for viral chromosome maintenance and transcription control, the precise mechanism through which CTCF and cohesin regulate KSHV latency transcription has not been elucidated.

CTCF is an 11-Zn finger DNA binding protein that binds to most of the characterized chromatin insulator elements in vertebrates [17], [18], [19]. CTCF can repress or activate transcription [20], [21], [22], prevent the spread of DNA methylation [21] and histone modifications [23], [24], [25], and block the interactions between transcriptional enhancers and promoters [26], [27]. CTCF has also been implicated in DNA looping and conformations that regulate long-distance chromosome interactions [28], [29]. Genome-wide mapping studies have found that CTCF colocalizes with certain histone modifications (e.g. H3K4me3) and histone variants (e.g H2AZ), as well as with cohesins at a high-percentage of binding sites [30], [31], [32], [33], [34]. Cohesins have a well-established role in mediating sister-chromatid cohesion [35], [36], and have also been implicated in developmental gene regulation [37]. Heritable mutations in human SMC1 and SMC3 result in a spectrum of developmental disorders collectively referred to as cohesinopathies, which include Cornelia de Langue syndrome [38], [39]. More recently, cohesins have been implicated in mediating promoter-enhancer interactions independently of CTCF binding sites, suggesting that their role in gene regulation is more extensive and complex than previously appreciated [40].

In this work, we investigate the role of CTCF and cohesins in KSHV chromatin conformation and the contribution of these factors to gene regulation and viral genome maintenance. We found that CTCF-cohesin mediates interactions between the 5′ end of the latency control region and its 3′ end. Remarkably, we also identified an interaction between the 5′ end of the latency control region and the promoter regulatory region of the lytic immediate early gene ORF50. We show that these interactions are important for the control of both lytic and latent gene transcription, and maintenance of genome copy number in latently infected cell populations.

## Results

### Chromosome conformation analysis of KSHV in a latently infected B-cell lymphoma cell line

**Figure 1:**
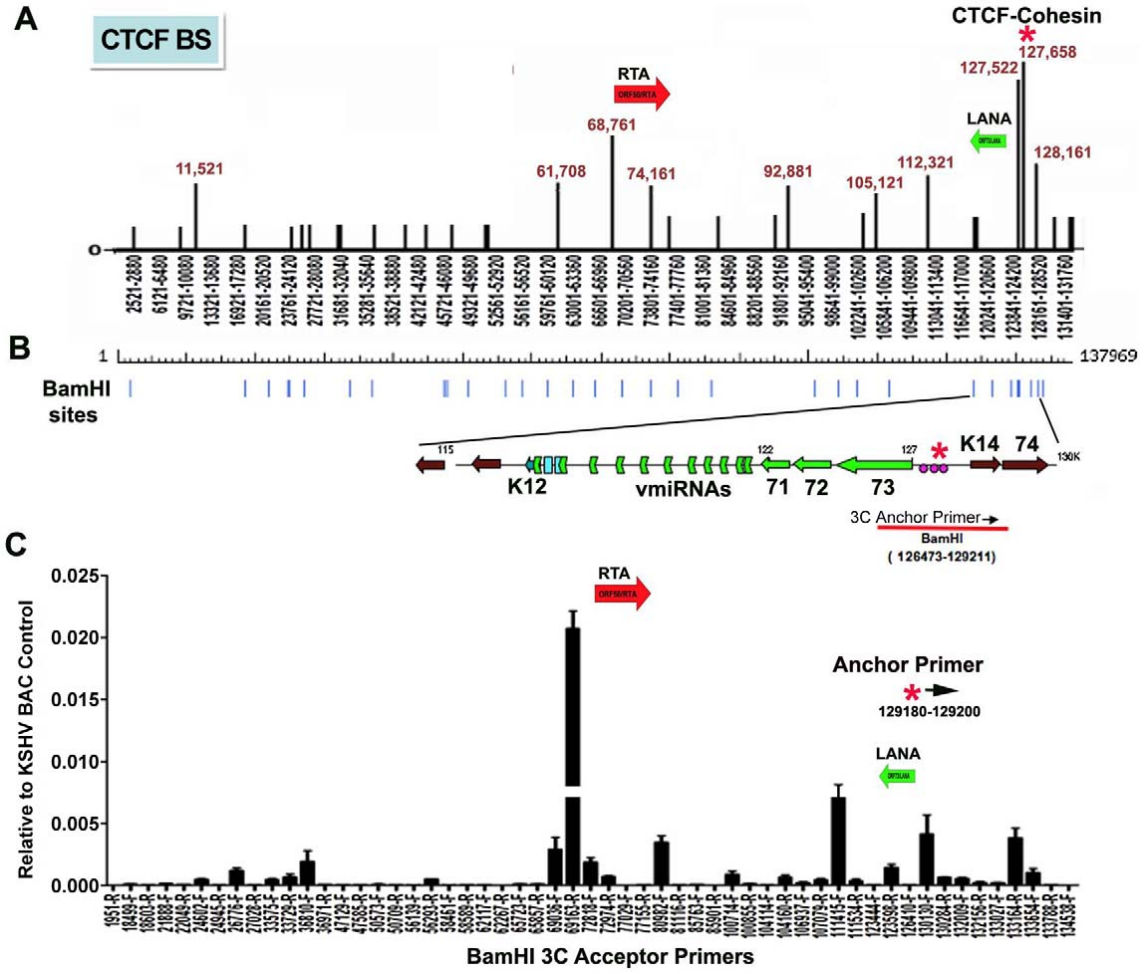
Chromatin conformation capture (3C) analysis of KSHV genome in BCBL1 latently infected B-cells. A) Schematic map of CTCF binding sites in the KSHV genome. Positions at 11521, 61708, 68761, 74161, 92881, 105121, 112321, 127522, 127658, and 128436 have been validated by real-time PCR analysis of CTCF ChIP DNA. Other sites are predicted by consensus and experimentally identified at lower resolution by genome-wide array analysis. CTCF-cohesin binding site is indicated by red asterisk. Positions of the Rta gene (red arrow) and LANA gene (green arrow) are indicated. B) Map of BamHI sites in the KSHV genome. C) Real-time PCR analysis of 3C ligation products between an anchor primer (KSHV region 129180–129200, 5′ primer) with BamHI acceptor primers as indicated in the X-axis. The acceptor primers were designed from 5′ and 3′ end of BamHI fragments throughout KSHV genome. PCR products were quantified relative to BamHI religation products from Bac36A control reactions. Error bars indicate the standard deviation from the mean for three independent 3C reactions.

CTCF binding sites have been mapped to various positions across the KSHV genome, including positions within the first intron of the latency transcript encoding LANA (KSHV coordinates 127,522-127,658 for genbank:AF148805.2), and upstream of the ORF50 promoter region (coordinates 68761) [15] (Fig. 1A). Cohesin subunits, SMC1, SMC3, and Rad21, were found to be highly enriched at the CTCF site within LANA intron (127,522) [15]. CTCF and cohesin are both implicated in higher-order DNA structure and looping. To determine if CTCF-cohesin binding at the latency control region mediated physical interactions with other KSHV regions, we used Chromosome Conformation Capture (3C) methods (Fig. 1B) [41], [42]. BCBL1 cells carrying latently infected KSHV episomes were cross-linked with formaldehyde and fragmented with BamHI, which generates 39 fragments of length varying from 31 to 16,659 bp. Fragments were diluted, religated, and then assayed by PCR for possible 3C ligation products. The 5′ end of the BamHI fragment from 126473-129211 that encompasses the CTCF-cohesin binding site was used for the anchor primer. We assayed PCR products between the anchor and the 5′ (F) or 3′ (R) ends of 28 other KSHV BamHI fragments spanning most of the KSHV genome (Fig. 1B). Ligation products were quantified by real-time PCR and normalized to ligation products generated from purified KSHV bacmid DNA random religation matrix [41]. Using this method, we found an enrichment of ligation products formed with the CTCF-cohesin anchor fragment (primer at 129211) and the region upstream of ORF50 (69163R). Ligation products were also enriched with neighboring primers (69036F and 72818R). Other ligation products were detected at 80982F, 111415F, 123598R, 130130F, and 133,164R. The ligation products formed between the anchor primer (129,211) with 3′ end of the latency transcript (111,480), as well as the promoter region of ORF50 (69163) were confirmed by DNA sequencing (Fig. S1). To further validate the PCR products were ligation dependent, we eliminated the ligation step from the 3C protocol (Fig. S2). In the absence of ligation, no PCR products were by amplified, indicating that the measured products are specific for 3C (Fig. S2).

To further investigate the intramolecular linkages of the CTCF-cohesin site in the KSHV latency control region, we used variations of two other chromatin conformation methods, namely circular chromosome conformation capture (4C) [43] and chromatin conformation capture carbon copy (5C) [44] (Fig. S3 and S4). For the 4C approach, BCBL1 or negative control Raji cells were cross-linked with formaldehyde and fragmented with Xma I, which generates a fragment from 127,591–127,756 that encompasses the CTCF-cohesin binding sites. After cross-linking and ligation, amplified linear fragments were generated by primer pairs for inverse PCR of captured circular DNA molecules. Control reactions also included non-ligated (mock) and non-cellular DNA derived from purified Bac36 (Fig. S3 panel A). Amplified DNA was detected only with KSHV positive BCBL1 cells (Fig. S3 panel B), and discrete DNA fragments were cloned and sequenced. We obtained eight clones with partial tandem duplication of CTCF binding cluster suggesting that self-ligation occurred frequently, perhaps reflecting aspects of sister-chromatid cohesion. We also obtained three clones containing Xma I fusions of the CTCF-cohesin (fragment 127434–128057) with the region 3′ of K12 (fragment 116650–117211). The clones identifying interaction with 3′ end of K12 suggest that CTCF may link the 5′ and 3′ ends of the latency transcription unit.

For the 5C approach, BCLB1 cells were cross-linked and digested with Nco I, which generates 64 KSHV fragments (Fig. S4). After religation, a library of fragments was amplified using inverse primers anchored at the CTCF-cohesin binding site region (126734–127590). The identity of the cross-linked DNA was then determined by real-time PCR using an array of 384 primers spanning the KSHV genome [13]. We found that the CTCF-cohesin region interacts with several other regions of the KSHV genome. Most notable was the major peak of self-ligation and amplification at the CTCF-cohesin binding sites (126734–127590). Consistent with 3C data shown in Fig. 1, we also detected a peak at the K12 3′ region (Nco I fragment 115311–115382). Similarly, we found strong peaks spanning the Rta promoter upstream of ORF50 (Nco I fragment 68673–70011). Several other minor peaks were detected which to map to known CTCF binding sites, suggesting that CTCF may mediate several intramolecular linkages on the KSHV genome. Taken together, these studies suggest that the KSHV forms a major linkage between the CTCF-cohesin binding sites in the latency control region and the 3′ end of the latency transcript, as well as with the ORF50 5′ promoter region.

### CTCF binding sites are essential for cohesin binding at the KSHV latency control region

**Figure 2:**
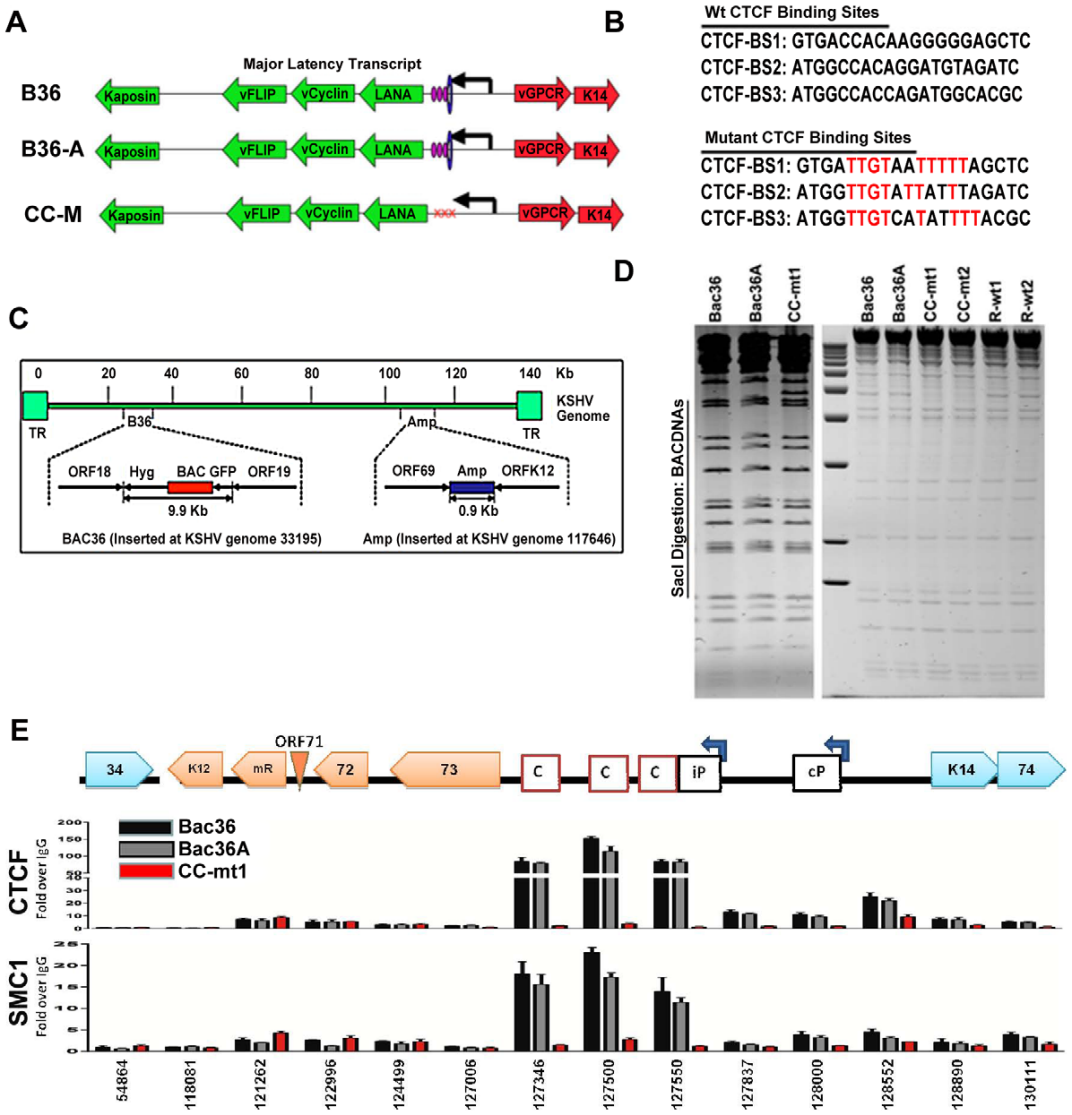
Construction of a KSHV bacmid genome lacking CTCF-cohesin binding sites in the latency control region. A) Schematic diagram of bacmids Bac36, Bac36A, and CC-M (and two independent versions named CC-mt1 and CC-mt2) and the relative position of CTCF and cohesin binding sites to latency transcripts. B) Sequence of three CTCF binding sites and the substitution mutations inserted to create CC-M mutant viruses, renamed CC-mt1 and CC-mt2. C) Schematic of Amp gene insertion used to stabilize the recombination within the latency control region. D) Restriction digest demonstrating the integrity of Bac36, Bac36A, CC-mt1, CC-mt2, and revertants R-wt1, R-wt2. E) ChIP assay across the KSHV latency control region demonstrating the CC-mt1 virus fails to bind CTCF and the SMC1 cohesin subunit. Genome positions are indicated above each bar graph.

**Figure 3:**
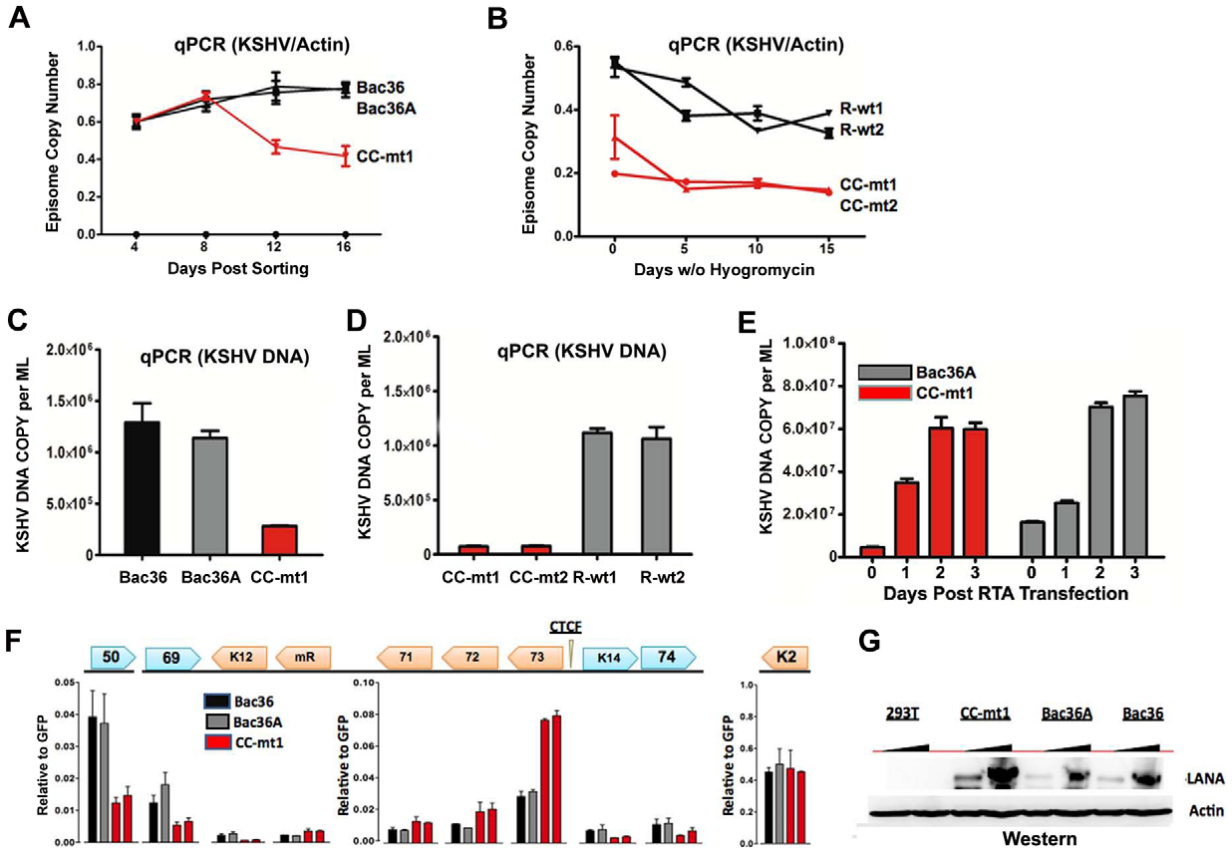
CTCF-cohesin reduces viral DNA copy number and deregulates latent and lytic gene transcription. A) Bac36, Bac36A, and CC-mt1 were transfected into 293T cells and sorted for GFP positive expression by FACS. KSHV bacmid DNA copy number was then assayed by qPCR at 4, 8, 12, and 16 days post-sorting and quantified relative to cellular actin. B) Hygromycin resistant 293T cell pools carrying CC-mt1, CC-mt2, R-wt1, or R-wt2 KSHV bacmid DNA were cultured in the absence of hygromycin selection and then analyzed at 0, 5, 10, and 15 days for relative KSHV bacmid copy number using qPCR of KSHV DNA relative to cellular actin. C) Extracellular viral DNA was quantified after TPA and sodium butyrate treatment of hygromycin resistant 293T cell pools containing Bac36, Bac36A, or CC-mt1, followed to be pelleted KSHV virion by ultracentrifugation and subjected to real-time PCR analysis. D) Same as in panel C, for extracellular viral DNA derived from CC-mt1, CC-mt1, R-wt1, and R-wt2. E) RT-qPCR analysis of KSHV transcripts (as indicated above) in 293T cells selected for Bac36, Bac36A, or CC-mt1 genomes. F) Viral DNA copy number was measured by real-time PCR for Bac36A (grey) or CC-mt1 (red) genomes in 293 cells transfected with Rta expression vector at 0, 1, 2, or 3 days post-transfection, as indicated. DNA was measured from hygromycin resistant cell pools. G) Western blot of LANA at two different concentrations from unselected or Bac36, Bac36A, or CC-mt1 selected 293T cells. Actin is shown as a loading control.

To determine if CTCF and cohesin binding sites were important for DNA linkages identified in the 3C assays, we used recombineering of KSHV bacmids to mutate each of the three CTCF binding sites in the latency control region (Fig. 2). Substitution mutations known to disrupt recombinant CTCF binding *in vitro* were first introduced into a plasmid containing the three CTCF binding sites (Fig. 3B) [16]. To facilitate efficient recombination, we generated a derivative of Bac36, termed Bac36A, that contains an ampicillin gene between K12 and ORF69 (Fig. 2C). The parent bacmid Bac36, Bac36A, and two independent but identical CTCF substitution mutations CC-mt1 and –mt2 were generated using recombineering, as were wild-type reversions of the CTCF mutants, R-wt1 (for CC-mt1) and R-wt2 (for CC-mt2). All bacmid clones were validated by restriction digest (Fig. 2D), analytical PCR, and DNA sequencing across the mutagenized regions (data not shown). To demonstrate that the substitution mutations in bacmids do indeed disrupt CTCF and cohesin binding in living cells, we used the chromatin immunoprecipitation (ChIP) assay (Fig. 2E). Primers spanning the latency control region were used to measure both CTCF and the cohesin subunit SMC1 for binding in hygromycin-resistant 293T cell pools containing either Bac36, Bac36A, or CC-mt1 genomes. We found that CTCF and cohesin binding were highly enriched at the previously established three CTCF binding sites in wt Bac36 and Bac36A, but completely eliminated from the CC-mt1 genome. A similar observation was made with other cohesin subunits SMC3 and Rad21 (data not shown). This result demonstrates that the substitution mutations disrupt CTCF binding, and that CTCF binding is necessary for cohesin binding in living cells.

### CTCF-cohesin binding sites regulate KSHV genome maintenance and lytic reactivation in 293 cells

To determine if the CTCF-cohesin binding sites contribute to viral genome maintenance, we introduced KSHV bacmids into 293T cells and selected with hygromycin followed by GFP cell sorting. The hygromycin resistant and GFP positive cells were than analyzed for stable maintenance of KSHV genomes after removal of hygromycin selection (Fig. 3). KSHV genomes were quantified by real time PCR analysis and normalized with primers specific for cellular actin (Fig. 3A and B). We first compared copy number for bacmids CC-mt1 with Bac36 and Bac36A-wt at 4, 8, 12, and 16 days post-sorting in the absence of hygromycin selection. We found similar genome copy numbers at 4 days post-sorting, but found that CC-mt1 genomes were reduced by 2-fold relative to Bac36 and Bac36A genomes by 16 days post-sorting (Fig. 3A). We also compared genome copy number for CC-mt1, CC-mt2, R-wt1, and R-wt2 in 293T cell pools after removal of hygromycin selection (Fig. 3B). We found that CC-mt1 and –mt2 had ∼2 fold fewer genome copies relative to R-wt1 and wt2 at all time points tested, indicating that the disruption of the CTCF binding sites in the KSHV latency control reduces episome copy number in 293T cell pools. This suggests that CTCF binding is important for maintaining stable genome copy number in latently infected cells.

We also tested the effect of CTCF deletion on lytic viral production using either chemical inducers TPA and sodium butyrate (Figs. 3C and D) or ectopic Rta transfection (Figs. 3E). We found that CTCF mutants (CC-mt1 and –mt2) yielded ∼10 fold less viral DNA than wild-type genomes (Bac 36, Bac36A in Fig. 3C, and R-wt1, or R-wt-2 wt in Fig. 3D) after induction with TPA and sodium butyrate. In contrast, when Rta transfection was used to induce lytic replication, we found no difference in virus production from wt and CTCF mutant bacmid genomes (Fig. 2E). These findings indicate that CTCF binding in the latency control region can regulate viral lytic replication through mechanisms that may involve Rta gene activation.

### Deregulation of latent and lytic transcription in genomes lacking CTCF-cohesin binding sites

The effect of CTCF mutation on gene expression was examined in stable 293 cell pools at 4 weeks after hygromycin selection (Fig. 3F). Gene expression for canonical latency transcripts (e.g. ORF73, ORF72, ORF71, miRNA, and K12), lytic transcripts (ORF50, ORF69, and K2), and neighboring transcripts observed in KS lesions (e.g. K14 and ORF74) were assayed by RT-qPCR relative to bacmid GFP and cellular actin. We found that latency associated transcripts for ORF73, 72, 71, and miRNA were elevated ∼2–3 fold in CC-mt1, relative to Bac36 and Bac36A. In contrast, transcription of K12, ORF50, ORF69, K14, and ORF74 were reduced by 2–3 fold by CTCF mutations. These results suggest that CTCF-cohesin binding sites in the LANA control region repress latency transcripts, but potentiates several lytic transcripts. The role of CTCF in limiting LANA expression could also be observed at the protein level by Western blot analysis (Fig. 3G). Similar patterns of gene expression were observed by primary infection of HUVEC cells with virus generated from Bac36, CC-mt1, and R-wt1 bacmid genomes (Fig. S5). This indicates that the CTCF-cohesin binding site is important for viral gene regulation in latently infected 293T cells and during primary infection of endothelial cells.

### CTCF-dependence of chromosome interactions

**Figure 4 ppat-1002140-g004:**
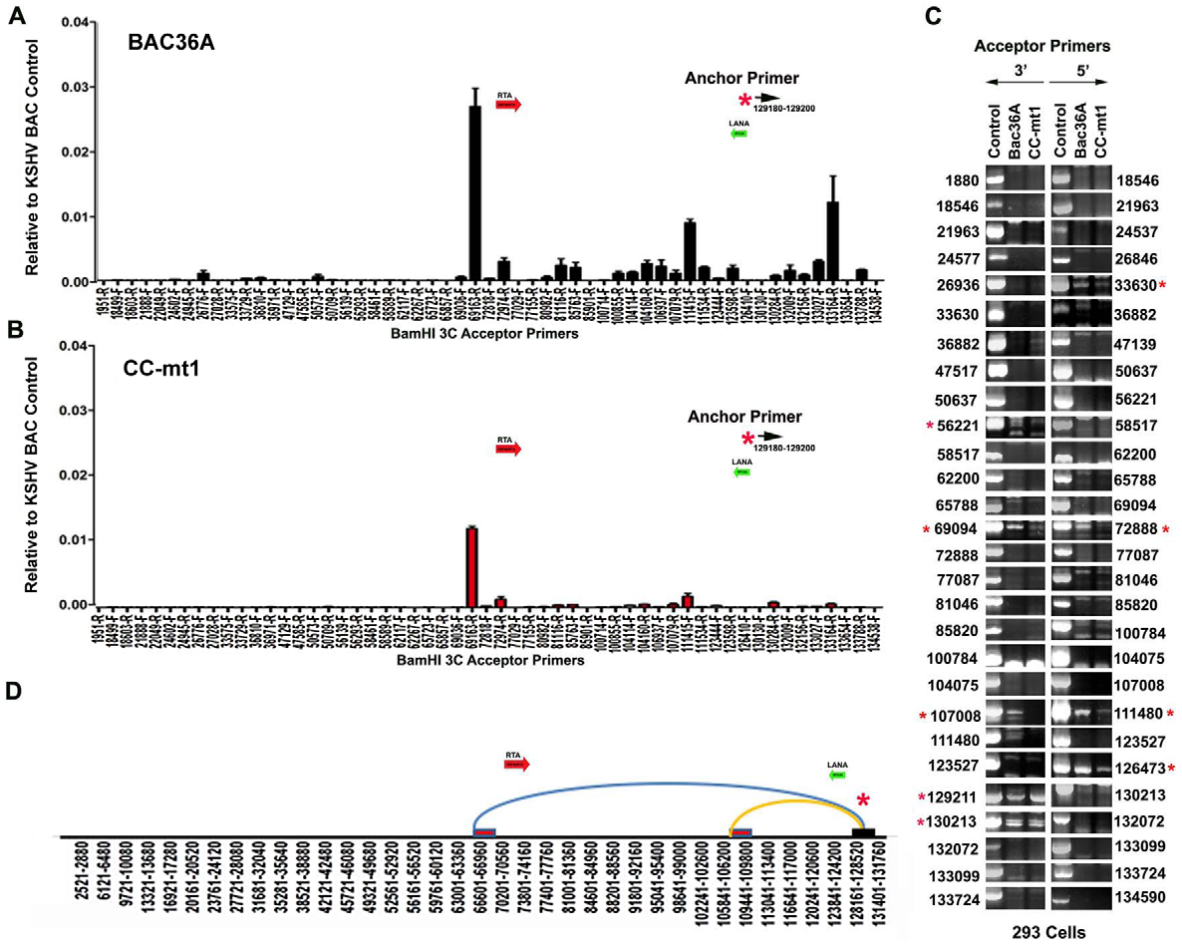
3C analysis of Bac36A-wt and CC-mt1 KSHV genomes in 293 cell pools. A) 293 cell pools containing Bac36A-wt genomes were assayed by 3C using methods essentially identical to that described for Fig. 1C. B) Same as in panel A, except for CC-mt1 genomes were used for 293 cell pools. C) Bac36-wt and CC-mt1 in 293 cell pools were analyzed for 3C using conventional PCR and a different set of primer pairs for each possible BamHI junction between the anchor primer (129020-129040) and acceptor primers for fragments as indicated. PCR products wee analyzed by agarose gel and ethidium staining. Control represents product generated with bacmid religation matrix. Numbers indicate BamHI cleavage sites and re-ligation positions. Red asterisks indicate formation of correct 3C ligation products. D) Summary of most frequent chromosome interactions determined by 3C assay.

To determine if any of the 3C chromosomal linkages were CTCF-dependent, we compared CC-mt1 and Bac36A-wt genomes in a 3C assay (Fig. 4). For these experiments, KSHV bacmid genomes from 293T cell pools were fragmented using Bam HI and assayed by real time PCR as described for Fig. 1. We found that the 3C pattern in Bac36A-wt containing 239 cell pools was nearly identical to that observed for KSHV in latently infected BCLB1 cells (compare Fig. 4A and 1C). In contrast, CC-mt1 genomes had reduced and fewer detectable linkages in 3C analysis (Fig. 4B). The linkage between the latency control region (128489F) and the 3′ end of K12 (107008R, 111480F) was reduced ∼7 fold, while the interaction with the Rta promoter (69161R) was reduced ∼2.5 fold relative to input DNA.

To further validate 3C linkages we generated a new set of primer pairs for agarose gel analysis of each BamHI ligation product formed with the anchor primer (Fig. 4C). KSHV DNA region 129020-129040 served as the new anchor primer. We compared 3C products from Bac36A-wt with CC-mt1 and compared these to control ligations generated with purified Bacmid under non-diluting conditions. Consistent with the real-time PCR analysis, we detected strong 3C ligation products between the anchor and 69094 (3′ primer), and between the anchor and 72888 (5′ primer). Specific PCR products were substantially more abundant in Bac36A-wt relative to CC-mt1 genomes. CTCF-dependent interactions were also observed between the anchor primer and 107,008 (3′ primer) and 111,480 (5′ primer), as well as with the anchor and 111,480 (3′ primer) (summarized in Fig. 4D). Additional interactions were observed between the 3′ anchor and 129,211 (3′ primer) and 130,213 (3′ primer), and with the 5′ anchor and 126,473 (5′ primer), but these interactions were less CTCF-dependent. No PCR products were detected in control samples that were not religated, indicating that these PCR products are 3C dependent (data not shown). Taken together, these results indicate that CTCF binding sites are important for the formation of several linkages between the latency control region and other regions of the KSHV genome.

### Cohesin and CTCF contribute to KSHV chromatin conformation and viral gene regulation in latently infected BCBL1 cells

**Figure 5:**
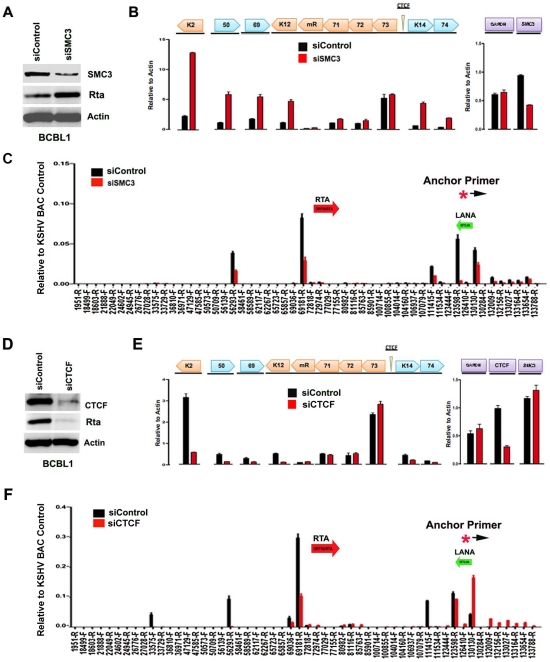
siRNA depletion of CTCF or Cohesin subunit SMC3 deregulates KSHV latent and lytic gene expression. A) Western blot of BCBL1 cells treated with siSMC3 or siControl, and probed with antibody to SMC3, Rta, and Actin, as indicated. B) siSMC3 (red) or siControl (black) treated BCLB1 cells were assayed by RT-qPCR for KSHV genes K2, ORF50, ORF69, K12, v-miRNA, ORF71, ORF72, ORF73, K14, and ORF74 (left) and cellular genes GAPDH or SMC3 (right) and quantified relative to cellular actin mRNA. C) 3C analysis of BCLB1 cells treated with siSMC3 (red) or siControl (black), using anchor primer 129180–129200, essentially as described in Fig. 1. D) Western blot of BCBL1 cells treated with siCTCF or siControl and probed with antibody to CTCF, Rta, and Actin, as indicated. E) siCTCF (red) or siControl (black) treated BCBL1 cells were assayed by RT-qPCR for KSHV genes, as described in panel B. F) 3C analysis for siControl (red) or siCTCF (black) treated BCBL1 cells, essentially as described in panel C, above.

The contribution of cohesin and CTCF proteins to KSHV conformation and gene regulation was investigated using siRNA depletion methods. BCBL1 cells were nucleofected with siControl or siSMC3, as well as with a GFP expression vector for FACS selection of transfected cells. GFP positive cells were then assayed by Western blot with antibody to SMC3, Rta, or Actin (Fig. 5A). We found that SMC3 was partially depleted (∼60%). We then assayed cells for RNA expression by qRT-PCR (Fig. 5B). We found that siSMC3 depletion had little effect on ORF73, ORF72, and ORF71 transcription, but caused a relatively large increase in several lytic viral genes, including K2, ORF50, ORF69, K12, K14, and vGPCR. SMC3 depletion caused an ∼60% reduction in SMC3 mRNA, consistent with Western blotting results. These findings suggest that SMC3 is important for the repression of KSHV lytic gene expression, including ORF50. siSMC3 and siControl treated cells were then assayed for 3C (Fig. 5C). We found that siSMC3 treated cells had reduced 3C linkages at several positions, including a ∼50% reduction in the linkage between the anchor primer (129180–129,200) and ORF50 control region (69181-R). An almost complete loss of 3C linkage was observed at position 123598. These findings suggest that SMC3 is important for these 3C linkages in BCBL1 cells.

We next assayed the effect of siRNA depletion of CTCF on KSHV gene expression and conformation in BCBL1 cells. Western blot analysis indicated siRNA depletion resulted in ∼80% loss of CTCF protein relative to siControl (Fig. 5D, top panel). Interestingly, CTCF depletion led to a corresponding loss of ORF50 protein (Rta) expression (Fig. 5D, middle panel). RT-PCR analysis further revealed that CTCF depletion resulted in a reduction in KSHV lytic gene transcription for K2 (∼8 fold), ORF50 (∼4 fold), ORF69 (∼3 fold), K14 (∼2.5 fold), and ORF75 (∼2 fold), with either no significant change in latency transcripts for ORF72 and ORF71, or with a slight increase in ORF73 latency transcript (Fig. 5E). 3C analysis revealed that CTCF depletion caused a reduction in the interaction between the anchor region (129180–129,200) and ORF50 control region (69181-R) (∼60% reduction). CTCF depletion caused a more significant loss of 3C signal at 111,415, suggesting that CTCF is critical for the link formed between the 5′ and 3′ ends of the major latency transcripts. A modest increase in 3C signal was observed with regions between 132–133kb, suggesting that this region of the viral chromosome may adopt an alternative conformation after CTCF depletion.

### Cell cycle-dependent chromatin conformation

**Figure 6:**
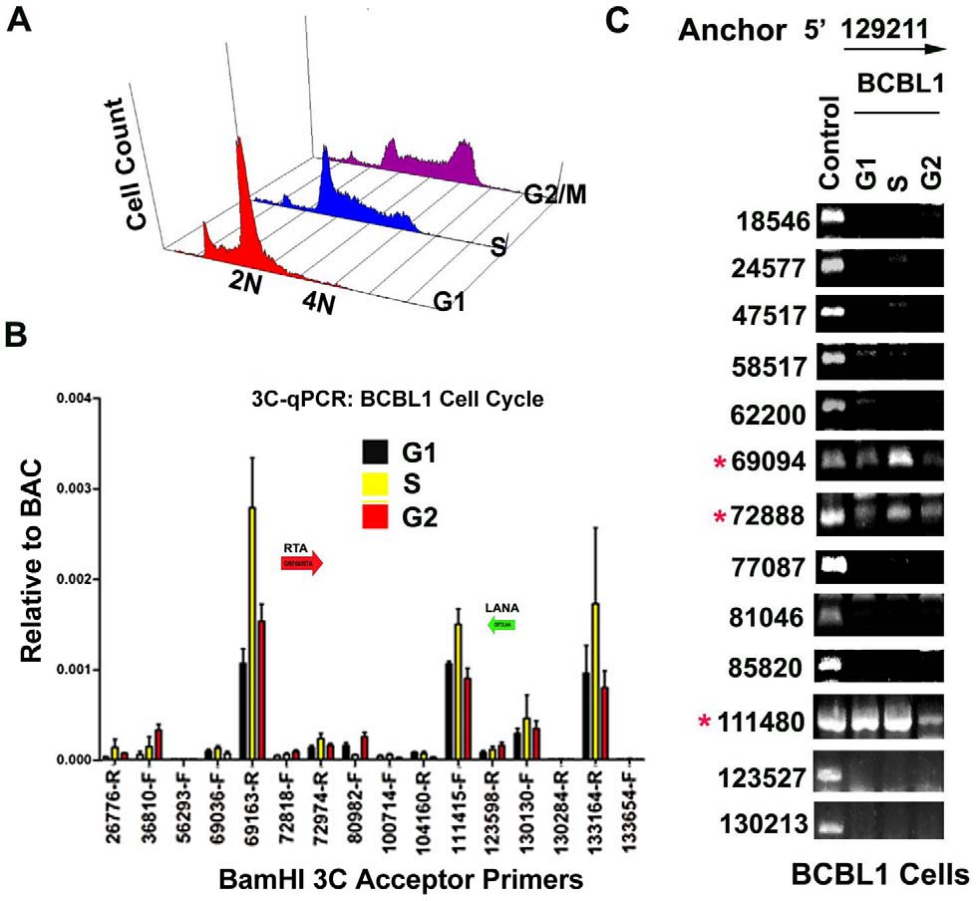
CTCF-dependent and cell-cycle dependent interactions between CTCF-cohesin and Rta (ORF50) promoter region. A) FACS analysis of cell cycle profile for BCBL1 used for cell cycle studies. Cells were fractionated by centrifugal elutriation prior to 3C processing. B) Real-time PCR analysis of 3C ligation products from BCBL1 cells enriched in G1 (black), S (yellow) or G2 (red). Anchor primer (129180–129,200) and acceptor primers are indicated on the X-axis, and essentially identical to those used in Fig.1. Error bars represent standard deviation from the mean for three independent PCR reactions. C) Conventional PCR analysis of 3C products for KSHV BamHI digestion using anchor primer 129020–129040 and acceptor primers for conventional PCR. Cell cycle dependent 3C products were assayed for G1, S, and G2 elutriated BCLB1 cells (as indicated above). Control is ligation products of Bac36A DNA (random ligation matrix). Numbers indicate BamHI sites and religation junctions. Red asterisk indicates formation of successful 3C ligation products.

In a previous study, we showed that cohesin and CTCF binding at the latency control region was cell cycle regulated, with a peak of cohesin binding in mid S phase [16]. We therefore assayed 3C at different stages of the cell cycle for BCBL1 cells (Fig. 6A). Cell cycle fractions were purified by centrifugal elutriation. 3C products were quantified by real-time PCR (Fig. 6B) or by agarose gel analysis of larger PCR products (Fig. 6C) and normalized to input DNA for each cell cycle condition. KSHV DNA regions 129020–129040 and 129180–129200 were used as the anchor primers for conventional (semiquantitative) and real-time PCR, respectively. We found that the interaction between CTCF-cohesin binding site and the BamHI sites at 69094 (3′ primer) and 72888 (5′ primer) were highly enriched in S phase relative to G1 and G2 phases using real-time PCR (Fig. 6B) or conventional PCR (Fig. 6C). The linkage at 111,480 (5′ primer) was also cell cycle regulated, with a large reduction in G2 (Fig. 6C). These findings are consistent with our previous study showing the cohesin association with CTCF is highly enriched in S phase, and indicate that the linkage between the latency control region and ORF50 promoter region (69094–728988), and the K12 3′ end (107008–111480) are cell cycle regulated in BCLB1 cells.

### Lytic cycle reactivation disrupts chromatin loop structure

**Figure 7:**
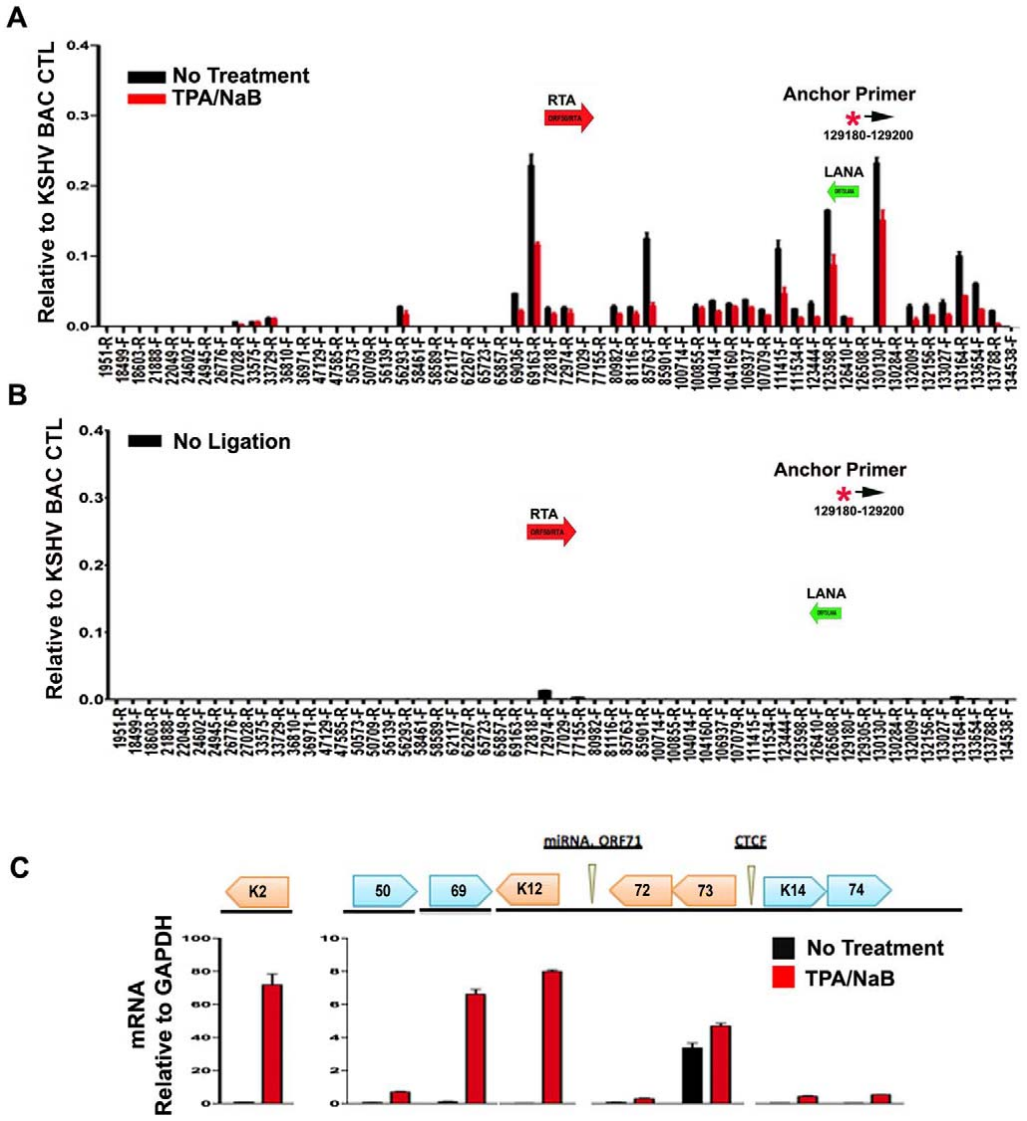
Loss of chromatin loops during lytic cycle reactivation. BCBL1 cells were untreated (black bars) or treated with TPA/NaB for 24 hrs (red bars). Cells were then assayed for 3C (panel A) or control 3C lacking ligation step (panel B) or RT-PCR analysis of RNA expression for KSHV K2, ORF50, ORF69, K12, ORF72, ORF73, K14, K74, and quantified relative to cellular GAPDH, as indicated (panel C). Anchor and acceptor primers for 3C analysis are essentially the same as that shown in Fig. 1.

To further explore whether these DNA linkages contribute to viral gene regulation, we performed 3C on latently infected BCLB1 cells shortly after induction of the viral lytic cycle (Fig. 7). KSHV lytic cycle was reactivated in BCBL1 cells by treatment with a combination of phorbol ester (TPA) and sodium butyrate (NaB) for 24 hrs. RT-PCR analysis was used to validate the viral lytic cycle gene expression was induced (Fig. 7C). As expected, viral lytic genes (e.g. K2, ORF69, K12) were highly induced (>50 fold) by TPA and NaB, while latency associated genes (e.g. ORF73 and ORF72) were only modestly increased (∼2 fold). ORF50 expression was also increased (∼8 fold). 3C analysis revealed that most of the previously observed linkages were reduced (∼50–75%) after TPA and NaB treatment (Fig. 7A). The linkages between CTCF-cohesin (129,180) and ORF50 (69,163) was reduced to 50% of untreated levels, while the linkage with K12 3′ (111,415) was reduced to near background levels. No significant 3C linkages were detected in control samples lacking DNA ligation (Fig. 7B). Since TPA and NaB stimulate reactivation in only ∼40–60% of the treated cells, the loss of 50% 3C signal is likely to reflect a substantial proportion of cells undergoing reactivation. Furthermore, 24 hr of TPA and NaB does not produce significant amplification of viral DNA since complete replication typically requires 48–72 hrs of treatment. Therefore, these data suggest that 3C linkages between latent and lytic control regions are largely disrupted during the early stages of lytic cycle reactivation.

## Discussion

Chromatin conformation has been implicated in mediating long-distance interactions between regulatory elements that control gene expression [18]. In this study, we examined the three-dimensional chromosome organization of the KSHV genome in latently infected cells. We found that the CTCF-cohesin binding site in the KSHV latency control region is in close physical proximity to the 5′ promoter region of ORF50, and to the 3′ end of the latency transcript (Fig. 1). Several different chromatin conformation capture methods were used to validate the linkages between these KSHV regions (Fig. S3 and S4). Linkages were further validated by using different primer sets for both real-time and conventional PCR, as well as DNA sequencing to verify the correct ligation junctions (Fig. S2). Chromatin conformation was disrupted by point mutations in the CTCF-cohesin binding site mutation (Fig. 4), as well as by siRNA depletion of CTCF or cohesin subunit SMC3 (Fig. 5). Importantly, disruption of CTCF and cohesin binding deregulated viral gene expression and copy number control (Figs. 3–5). Finally, we show that chromatin conformation is cell cycle-regulated (Fig. 6) and disrupted during viral reactivation from latency (Fig. 7). These findings suggest that CTCF and cohesin interactions regulate viral gene expression and episome stability through structural changes in viral chromosome conformation.

Herpesviruses exist in a dynamic equilibrium between latent and lytic gene expression programs. Cross-talk between latent and lytic gene regulatory factors have been described in both negative and positive feedback loops. For KSHV, both LANA and v-miRNAs have been implicated in down-regulating lytic reactivation, and Rta has been implicated in activating transcription of some latency gene products [13], [45], [46], [47], [48], [49]. For the gammaherpesviruses, some lytic gene expression is commonly observed among a population of latently infected cells. Some lytic gene expression and DNA replication may be required for maintaining viral genome copy number and persistence in a latently infected cell population [50]. Therefore, effective maintenance of viral genomes requires a coordinate regulation of both latent and lytic gene expression programs.

To better understand the epigenetic regulatory mechanisms that orchestrate latent and lytic gene expression programs, we investigated the chromatin architecture of the KSHV genome in latently infected cell populations. We used four independent methods to demonstrate that the CTCF-cohesin binding site at the LANA 5′UTR is in close proximity to the 3′ region of K12, and the promoter regulatory region of ORF50. Genome-wide 3C using BamHI fragments revealed a robust interaction with 5′ end of the latency control region (∼129,192) with the 3′ end of K12 (∼111,485), as well as with the promoter regulatory region of ORF50 (∼69,094). This interaction was observed in both BCBL1 cells (Fig. 1, 5, and 6) and in 293T cells carrying KSHV BACs (Fig 4). The interaction was also observed with different primer sets using either real-time or conventional PCR. In addition to conventional 3C, we identified an interaction between the 5′ and 3′ ends of the latency control region using circular chromatin conformation capture (4C) with XmaI digestion products (Fig. S3). Nearly identical interactions were also observed when the KSHV genome was fragmented using NcoI and 3C ligation products were assayed by genome-wide array after inverse PCR (Fig. S4). This method revealed strong interactions between the CTCF-cohesin binding region and the 3′ end of K12, as well as with positions upstream of ORF50 (Fig. S4). Several additional interactions were also observed, but their significance is not yet known. The interaction between CTCF-cohesin and the 3′ end of K12 was most consistently observed in all four methods. The interaction between CTCF-cohesin and the ORF50 promoter region was sensitive to mutation in the CTCF binding sites (Fig. 4), siRNA depletion of CTCF or SMC3 (Fig. 5), and was subject to cell cycle (Fig. 6) and KSHV lytic cycle (Fig. 7) regulation in BCBL1 cells. A previous study revealed that cohesin binding to the latency control region was cell cycle regulated, with occupancy peaking in S phase [16]. Lytic gene transcription was also found to be cell cycle regulated, with ORF50, ORF45, and vGPCR expressed at highest levels in G2 phase (Fig. S6 and [16]). Taken together, these studies indicate that CTCF and cohesins mediate a functional interaction between ORF50 promoter and the latency control region. The dynamic cell cycle regulation of this interaction suggests that lytic genes are partially activated each cell cycle. Since full lytic replication rarely occurs in these cells, the lytic cycle must remain blocked at additional steps in the reactivation process. The nature of these blocks to lytic replication remain obscure.

**Figure 8:**
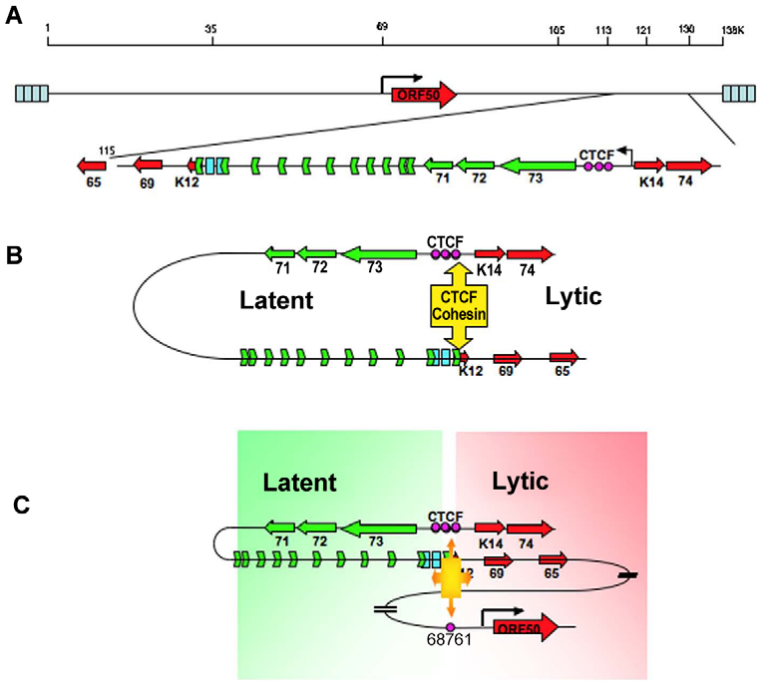
Model of CTCF-cohesin mediated chromatin conformation of KSHV latent episomes. A) Linear schematic of KSHV latency control region and major latency transcripts. B) Depiction of 3C predicted loop between the CTCF-cohesin binding site and the 3′ end of the major latency transcript. C) Depiction of 3C predicted higher-order interaction between CTCF-cohesin site and the ORF50 promoter control region. Speculative model of how CTCF-cohesin may function to both insulate and coordinate latent and lytic viral gene expression.

Long distance regulatory interactions, like those between promoters and enhancers, are thought to be mediated by chromatin loops [40], [51]. Several lines of evidence suggest that the chromatin loop formed between the CTCF sites in the latency control region and the ORF50 promoter regulatory region is functional in gene regulation. Mutations in the CTCF binding sites within latency control region caused a reduction in lytic gene transcription, including a 2–3 fold reduction in ORF50 mRNA located ∼60kb distance apart (Fig. 3). This corresponded to a 2–3 fold reduction in loop formation as measured by 3C (Fig. 4). CTCF site mutation or CTCF siRNA depletion reduced, but did not completely abrogate the loop formed between the latency control region and ORF50 promoter region. This suggests that other factors, in addition to CTCF, may also contribute to this chromatin loop. It is also possible that regulation of lytic transcription by CTCF binding site disruption may be partially explained by the indirect effects of latency gene products, like LANA and v-miRNA, on KSHV lytic transcription control. Both LANA and v-miRNA have been shown to down regulate ORF50 expression and reactivation function [13], [46], [52]. However, the large increase in lytic gene expression observed in BCLB1 cells after siRNA depletion of SMC3 (Fig. 5A), suggests that looping plays a major role in ORF50 transcription control. Interestingly, siRNA depletion of CTCF resulted in a loss of lytic gene transcription, while siRNA depletion of SMC3 resulted in an increase in lytic gene transcription. This indicates that the regulation by CTCF and cohesin can be uncoupled and antagonistic. Cell cycle regulated cohesin binding may modulate CTCF linkages that alter chromosome conformation. We propose that these changes in viral chromatin conformation coordinate changes in latent and lytic gene expression programs (Fig. 8).

Numerous studies indicate that CTCF plays a primary function in mediating inter- and intra-chromosomal linkages important for gene regulation and chromatin domain insulation [18], [28], [32]. Colocalization of cohesins with CTCF sites provides a mechanism for physical linkages of DNA elements, since cohesins are known to mediate sister-chromatid cohesion and long-distance regulatory interactions [40], [53], [54]. Because KSHV genomes are multicopy, our data does not completely distinguish between inter- and intra-molecular interactions. Inter-molecular interactions that may mediate sister-chromatid cohesion would be important to further characterize, and 4C data suggest that some of these sister-interactions may be occurring at the CTCF-cohesin site (Fig. S3). Nevertheless, CTCF binding sites are located at many of the KSHV 3C linkage sites, including those at the 3′ end of K12 (112,321), and the ORF50 promoter region (68761). These CTCF-dependent linkages are more likely to mediate intra-molecular loops that may provide transcription regulatory functions, including chromatin domain structure and insulator properties (Fig. 8). The latency control region appears to be organized into an actively transcribed loop containing the major latency cluster. This loop may form an active chromatin domain that can be linked to the ORF50 promoter in a cell cycle-dependent manner mediated by cohesin-CTCF interactions. In this way, chromatin loops may also provide insulator functions commonly attributed to CTCF.

Recent studies have shown that KSHV lytic cycle gene expression is under the control of “bivalent” histone modifications, similar to those described for genes important for embryonic stem cell maintenance [12], [14], [55]. Based on the data from these genome-wide epigenetic studies, the CTCF binding sites at the ORF50 promoter and latency control regions are generally spared DNA methylation [12], but are enriched for H3K4me3 and H3K27me3 [12], [14]. Both regions are enriched for polycomb group protein EZH2, which bind to H3K27me3 [14]. Interestingly, EZH2 and polycomb group proteins have been implicated in chromatin loop formation at cellular CTCF sites and at bivalent genes [56], [57], [58]. While we have not demonstrated the role of polycomb group proteins in the formation or regulation of CTCF-cohesin mediated loops, it will be important to determine if the EZH2 or H3K27 methyltransferases play a direct role in regulating KSHV chromatin loop formation. Dynamic loop formation between the latent and lytic regulatory regions may be an additional feature of the bivalent histone modifications that maintain genes poised for transcription activation. Future studies will be required to understand the full significance of chromatin architecture on viral gene expression, as well as the factors that determine the loop connections and movement in complex biological processes. We conclude that CTCF-dependent loop formation is likely to be a general mechanism utilized by many persistent DNA viruses, especially herpesviruses, that need to coordinate a dynamic balance between latent and lytic gene expression programs.

## Methods

### Cells

The KSHV-positive PEL cell line BCBL1 was cultured at 37°C and 5% CO_2_ in RPMI medium (Gibco BRL) and supplemented with 10% fetal bovine serum and penicillin-streptomycin (50 U/ml). The 293T human embryonic kidney cell line (ATCC) was maintained in Dulbecco's modified Eagle's medium supplemented with 10% fetal bovine serum containing penicillin-streptomycin (50 U/ml). Human umbilical vein endothelial cells (HUVEC) were maintained in EGM-MV microvascular endothelial cell growth medium (cc-3125; Lonza). The 293T-derived cell lines were all cultured identically to the 293T cells, except with the addition of 200 µg/ml hygromycin B for the selection of the wild-type (WT) and recombinant viral genomes.

### Chromatin conformation capture

Chromosome conformation capture experiments were carried out essentially as described in Vassetzky et al [59] with minor modification. 5×10^7^ 293T cells or 1×10^7^ BCBL1 cells cross-linked with 2 % formaldehyde at room temperature for 10 minutes. Cross-linking reactions were terminated by addition of glycine at the final concentration of 125 mM. Nuclei of crossed linked cells were purified by incubating the cells with lysis buffer (10 mM Tris-HCl, pH 7.5; 10 mM NaCl; 5 mM MgCl_2_; 0.1 mM EGTA; 1 × complete protease inhibitor; 11836145001 Roche) for 10 minutes on ice, douncing cells 10 times on ice, additional incubating on ice for 10 minutes, and finally centrifuging for 5 min at 400 *g* at 4°C. Resultant nuclei were resuspended in 0.5 ml of 1.2X restriction enzyme buffer and digested with 1500 units of BamHI overnight at 37°C. To inactivate the restriction enzyme, the digested samples were treated with 40 µl of 20% SDS at 65°C for 25 minutes and 70°C for 10 minutes. The samples were diluted in 6.125 ml of 1.15X ligation buffer and ligated with 150 units of T4 DNA ligase at 16°C for 5 hours and additional 0.5 hour at room temperature. Ligated samples were treated with 30 µl of 10 mg/ml Proteinase K at 65°C overnight to reverse cross-linking, and treated with 30 µl of 20 mg/ml Rnase A at 37°C for 1 hour. Genomic DNA was extracted with phenol/chloroform and precipitated with 3 M sodium acetate (pH 5.6) and EtOH. Precipitated DNA was washed with 70% EtOH and resuspended with 150 µl of 10 mM Tris (pH 7.5). Random ligation matrix was prepared as follows. 20 µg of KSHV BAC36 DNA was digested by 50 units of BamHI used for previous 3C analysis for 3 hours at 37°C. These digestion solution was twice extracted with phenol/chloroform, followed by addition of 3 M sodium acetate (pH 5.2) and ethanol precipitation at −70°C overnight. Precipitated DNA was dissolved in 1X ligation buffer and subjected to 20 units of T4 DNA ligase for 4 h at 16°C. Ligation solution was extracted with phenol/chloroform and precipitated with EtOH overnight at −70°C. Finally, the precipitated DNA pellet was dissolved in 100 µL of 10 mM Tris-HCl (pH 7.5). The random ligation matrix was used as KSHV BAC control in semi-quantitative PCR and Realtime PCR analysis.

### 4C

Circular chromosome conformation capture (4C) was performed essentially the same as with 3C, except that DNA from BCLB1 and KSHV negative control (Raji) cells was digested with XmaI and ligation products were amplified with primers inversely oriented from the XmaI fragment (127,434–128,057) encompassing the CTCF-cohesin binding sites. Bac36 DNA also served as an additional negative control for 4C. After ligation, primers for inverse PCR amplification were positioned at 127,525 (leftward) and 127,651 (rightward), and amplified 30 cycles with Taq polymerase. PCR products were analyzed first by agarose gel electrophoresis, and major PCR products were cloned using TA cloning and subject to DNA sequencing.

### 5C

Chromosome conformation capture carbon copy (5C) was performed essentially the same as with 3C, except that BCBL1 cross-linked DNA was first digested with NcoI and then subject to ligation. Ligation products were then amplified with inverse primers for the Nco I fragment encompassing CTCF-cohesin binding site (126734–127590). PCR was amplified for 30 cycles and purified to remove anchor primers. Purified PCR amplified DNA was then used as template DNA for an additional round of PCR amplification using a 384-well genome-wide array for KSHV primers, described previously [13], [15]. Ct values were normalized to Bac36 DNA for each primer set of the 384-well array and presented as the average value for 3 independent experiments.

### PCR analysis of 3C-BamHI products

Semiquantitative PCR was used to quantify linkages between KSHV BamHI fragments linked by 3C methods to the BamHI fragment containing the major latency control region. Each 25 ul of semiquantitative PCR reaction was composed of 100 ng (for BCBL1 cell) or 200 ng (for 293T cell) of genomic DNA. A gradient PCR program used was to optimize for the various primer pairs. Briefly, PCR amplification included 1 cycle for 95°C for 5 min, 28 (BCBL1 cells) or 35 (293T cells) cycles for 95°C for 30 sec, gradient annealing temperature for 1 min, 72°C for 1 min, 1 cycle for 72°C for 10 min. Gradient annealing temperature was set from low melting temperature (TM) 51°C to high TM 58°C of paired primers for 3C products. KSHV BAC control (random ligation matrix) was included in parallel in all PCR analysis. Realtime PCR was also used for quantifying 3C ligation products. For realtime PCR analysis, 50 ng (BCBL1 cells, KSHV BAC control) or 100 ng (293T cells) of 3C products were used in 12.5 µl of realtime PCR reaction and quantified in 96 or 384 well formats using ABI instruments. Relative quantification for each primer set was obtained from Ct valued based standard curve method. Resultant quantification of 3C products was divided with that of KSHV BAC control and compared among different primer sets. Each primer sets (spanning most of the major BamHI fragments of the KSHV genome) were designed to produce less than either 1 kb (semiquantitative) or 0.1 kb (Realtime) PCR products that span a BamHI digestion site corresponding to the primer set. The anchor primer for real-time PCR was positioned at 129180–129200. Semiquantitative PCR or realtime PCR primer sequences used for 3C analysis are provided in supplemental data (Tables S1–S4).

### Genetic manipulation of KSHV BAC construction

Mutagenesis of BAC36 was performed with Counter-Selection modification kit as recommended by manufacturer (Gene Bridges). To facilitate recombination at the KSHV latency locus, the ampicillin resistant gene from pGEM-T vector was introduced at KSHV genome at 117640 bp by homologous Red/ET recombination, then used as second selection marker along with chloramphenicol in the subsequent recombination process. This increased recombination efficiency >500 fold. Resultant KSHV BAC was named BAC36Amp (Bac36A), which was used for site-directed mutagenesis in the three CTCF/Cohesin binding sites (127300–127500) by the two-steps homologous Red/ET recombination. In brief, control region of KSHV major latency transcript was replaced with rpsL-neo counterselection/selection cassette in first round Red/ET recombination. The rpsL-neo cassette was then swapped with a non-selectable KSHV DNA carrying site-directed mutation in CTCF binding sites throughout second round recombination. The substitution mutations in each of the three CTCF binding sites was first generated in a plasmid that has been described and characterized previously[16]. The plasmid containing the three CTCF substitution mutations was used for PCR amplification for homologous recombination into the KSHV bacmid. Two independent, identical mutants (CC-mt1 and CC-mt2) were generated and confirmed by PCR, restriction digestion, and sequencing of the insertion junctions. The CTCF substitution mutation was described in plasmid form previously [16]. Revertants of CC-mt1 and CC-mt2 were generated by re-integrating rpsL-neo, followed by replacement with wt CTCF binding sites. The resulting Bacmids R-wt1 and R-wt2 were confirmed by PCR, restriction digest, and DNA sequencing across the insertion region.

### Quantification of intracellular and extracellular KSHV genomic DNA

To quantify intracellular KSHV DNA copy number, 293 cells (use approximately 1×10^6^ cells per sample) carrying KSHV BACs were collected and resuspended in 100X SDS lysis buffer (1% SDS, 10 mM EDTA, 50 mM Tris, pH 8.0.). After brief sonication, immunoprecipitation (IP) dilution buffer (0.01% SDS, 1.1% Triton X-100, 1.2 mM EDTA, 16.7 mM Tris, pH 8.0, 167 mM NaCl) was added to 1 ml and then incubated with proteinase K for 2 to 3 h at 50°C. Cell lysate (300 µl) was removed and subjected to phenol-chloroform extraction and ethanol precipitation. Precipitated DNA was then assayed by real-time PCR using primers for ORF50 or ORF69 and normalized by the cellular DNA signal at the actin or GADPH gene locus. To measure extracellular KSHV genomic DNA copy number, 10×10^6^ cells were seeded on 100-mm dish plate in 10 ml of DMEM. The next day, the KSHV lytic replication was induced by adding TPA (20ng/ml) and sodium butyrate (1 mM) for 3 additional days. The induced culture media were collected and filtered through 0.45-µm filters. The virions were then pelleted by ultracentrifugation on a 25% sucrose cushion at 100,000 x *g* for 1 h with a Beckman SW28 rotor. The pellets were dissolved in 200 µl of 1 X phosphate-buffered saline (PBS), treated with DNase I at 37°C for 1 hr, followed by Proteinase K treatment at 56°C for 20 min, extracted virion DNA with Phenol/Chloroform/Isoamyl alcohol solution (PCI), and finally precipitated with EtOH. Quantification of resultant virion DNA was determined by Real-time PCR. Real-time PCR was conducted with either primers such ORF50 or ORF69 and normalized by the cellular DNA signal at the actin or GADPH gene locus. Titration of 4.882 pg – 0.009 pg of KSHV BAC36 DNA was used to obtain a calibration curve using primers specific to KSHV genes (ORF50 or OR69) or cellular genes (beta-Actin or GAPDH).

### Viral infection of HUVECs

Freshly prepared BAC DNAs (BAC36, BAC36Amp, BAC-CC-mt1, BAC-CC-mt2, BAC-R-wt1, BAC-R-wt2) were introduced into 293T cells by transfection. Briefly, 2 µg of the respective BAC DNA was transfected into 293T cells grown to a 40–60% confluence in a 60-mm dish with the QIAGEN Effectene transfection kit. Forty-eight hours post-transfection, the transfected cells were subcultured into 100-mm dish plate with fresh medium containing 200 µg/ml hygromycin (Roche). In approximately 10–14 days post-transfection, hygromycin-resistant clones were pooled together and transferred into a new 150-mm dish plate. When the monolayer reached 80–90% confluence, the cells carrying KSHV BACs were split into new 150-mm dish plate to be maintained by periodic splitting. To obtain KSHV virions, cells were induced with 20 ng/ml TPA and 0.3 mM sodium butyrate. Typically, ∼20 150-mm dish plate were induced for 4 to 5 days, and released virion particles were purified from the supernatant. The induced culture media were collected and filtered through 0.45-µm filters. The virions were then pelleted by ultracentrifugation on a 25% sucrose cushion at 100,000 x *g* for 1 h with a Beckman SW28 rotor. The pellets were dissolved in 1% of the original volume of 1 X phosphate-buffered saline (PBS) or Dulbecco's modified Eagle's medium and stored at 80°C. HUVECs at 70% confluence on 12-well plates were infected with KSHV virion diluted in EGM-2 containing 4 ug/ml polybrene under spinning at 2,500 rpm for 1 h at room temperature. Cells were washed twice and EGM medium was changed every day until assay was conducted.

### ChIP

Chromatin immunoprecipitation (ChIP) was performed according to the protocol by Upstate Biotechnology Inc., and as previously described [16]. Diagenode Bioruptor was used to sonicate genomic DNA into 200- and 400-bp DNA fragments according to manufacturer's protocol. Resultant cell lysates were subjected to immunoprecipitation with indicated antibodies, then followed by real-time PCR analysis to determine the final ChIP products.

### siRNA treatment

siRNA targeting CTCF and SMC3 was purchased from Dharmacon RNAi Technologies; CTCF siGENOME siRNA designed from 3′-UTR (D-020165) and SMC3 siGENOME siRNA designed from ORF (D-008738). 5 ul of 40 nM siRNA solution and 1000 ng of pGmax-GFP vector were nucleofected into 5–10 million BCBL1 cells using 100 ul of Cell Line Nuclefector solution V (Amaxa Nucleofector Technology). S-02 program was used in Amaxa Nucleofector system. In parallel, ON-TARGET plus Non-targeting siRNA #1(D-001810-01) was nucleofected into BCBL1 cell for control. In 2–3 days post nucleofection, GFP positive BCBL1 cells were sorted and then used in RT-qPCR, Western Blotting and 3C analysis. RT-qPCR and Western Blotting analysis confirmed loss of either CTCF or SMC3. AntiLANA antibody was purchased from Advanced Biotechnology and antiRTA antibody was from gift of Dr. Yuan and Dr. Robertson.

## Supporting Information

Figure S1 **Sequence of 3C ligation products formed between latency control regions (129216F) and ORF50 promoter (69094R) or 3′ end of latency transcript (111485R).** Sequencing chromatogram for 3C generated PCR products amplified with primers 129216F and 69094 (top panel) or with 129216F and 111484R (lower panel).
(TIF)

Figure S2 **No Ligation Control for 3C.** BCBL1 cells were treated essentially identically to cells treated in experiments shown in Fig. 1, but ligase was eliminated from the 3C protocol. These results show that all 3C signals are dependent on the ligation reaction.
(TIF)

Figure S3 **Circular Chromosome Conformation Capture (4C) of CTCF-cohesin site.** A) Schematic of KSHV latency control region and XmaI restriction fragments. Anchor primers within 127434-128057 were used for inverse PCR and nested PCR amplification. B) Fragments amplified from 4C experiment with either mock (non-ligated DNA), purified Bac36 DNA, Raji cell, or BCBL1 cell DNA were fractionated by agarose gel electrophoresis. M is 1 kB DNA ladder. Arrows indicate fragments clones into TA-cloning vector. C) Schematic depiction of 3 clones containing CTCF-cohesin XmaI site fused to the K12 3′ end (Xma I site at 117211). D) Schematic depition of 8 clones showing a tandem duplication of the CTCF-cohesin binding site indicative of self-interactions.
(TIF)

Figure S4 **KSHV genome-wide PCR array analysis of chromatin conformation capture carbon copy (5C) products with anchor at CTCF-cohesin site.** BCBL1 cells were processed for 3C analysis using NcoI to fragment the genome and inverse PCR primers anchored around the CTCF-cohesin binding site at 127,450. 3C amplified products were then quantified by real-time PCR using a KSHV genome-wide array. Peaks are indicated at the anchor site, K12 3′ end, Rta (ORF50) promoter, and position 99803.
(TIF)

Figure S5 **CTCF-cohesin site is required for control of latent and lytic transcription in primary infection of HUVEC cells.** A) HUVEC cell infection with mock, CC-mt1, or R-wt1 bacmid derived virus assayed for GFP at 24 hr or 48 hrs, or for KSHV ORF45 at 48 hrs post infection. B) Intracellular viral DNA copy number in HUVEC infected cells at 48 hrs post infection virus from Bac36, R-wt1 or CC-mt1 genomes. C) RT-PCR of KSHV gene expression relative to cellular actin for HUVEC cells infected with Bac36 (black), R-wt1 (gray), or CC-mt1 (red). KSHV genes are indicated above each bar graph.
(TIF)

Figure S6 **Increase in lytic gene expression in G2/M cells.** BCBL1 cells were fractionated by centrifugal elutriation as described for Fig. 6. Cells from the G1 phase (18 ml/min) or G2/M phase (46 ml/min) were mounted by cytospin, fixed with paraformaldehyde, counterstained with DAPI, and then assayed for indirect immunofluorescence with antibodies to ORF45 (green), or histone H3 phosphoS10 (H3pS10) (red). These findings show that the majority of G2/M cells, as indicated by H3pS10 positive, are also positive for ORF45. This indicates that lytic cells are not a small subfraction of those cells elutriated during G2/M.
(TIF)

Table S1 **Primers for 3C assay using real-time PCR.**
(XLS)

Table S2 **Primers for 3C assay using conventional PCR.**
(XLS)

Table S3 **Primers for RT-PCR assays.**
(XLS)

Table S4 **Primers for ChIP assays.**
(XLS)
