# Supplementary figures and images for: Coordination of KSHV Latent and Lytic Gene Control by CTCF-Cohesin Mediated Chromosome Conformation
Source: PLoS Pathog. 2011 Aug 18;7(8):e1002140. doi: 10.1371/journal.ppat.1002140 (PMC3158054; doi:10.1371/journal.ppat.1002140)

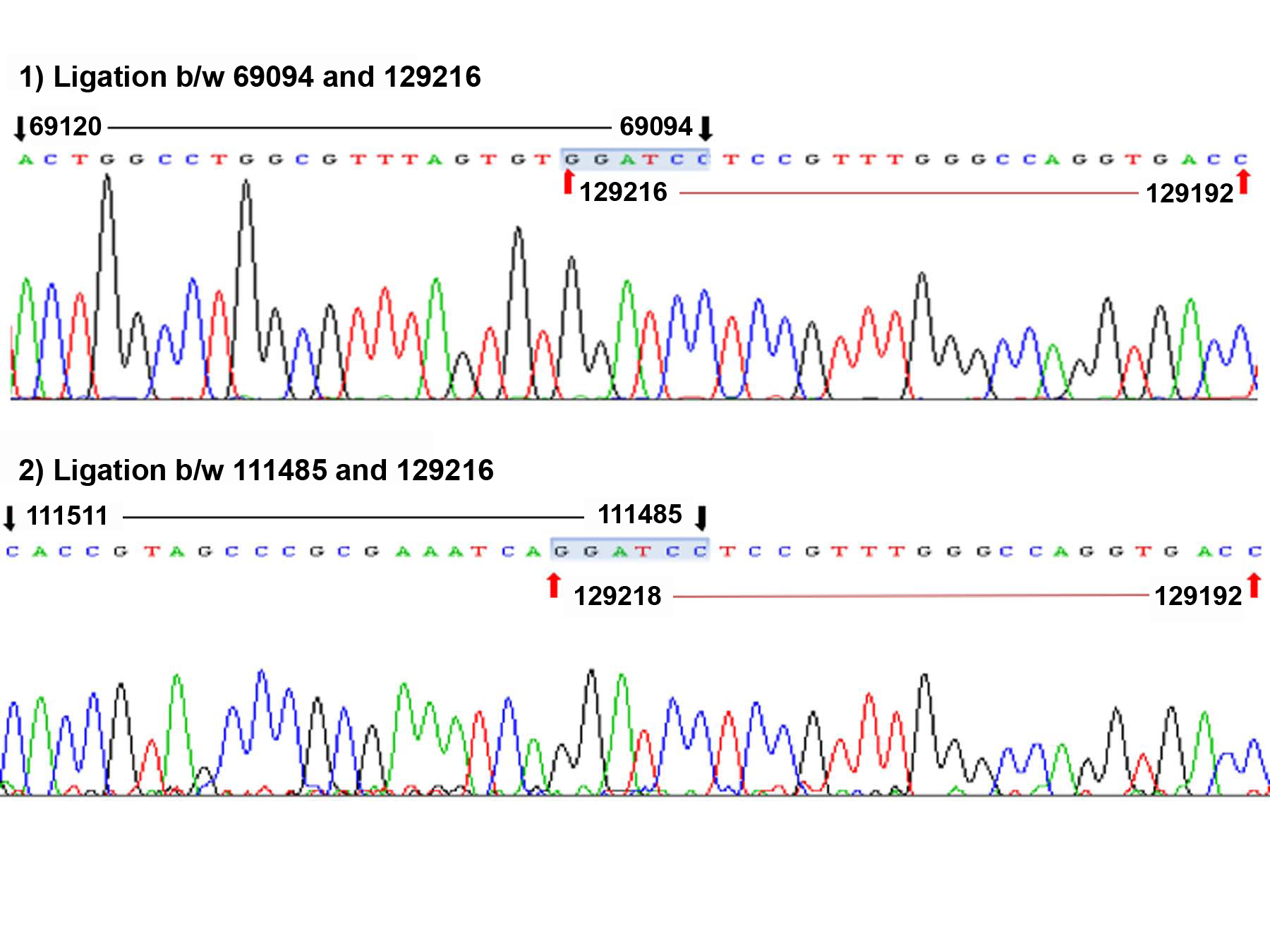

Supplement: Figure S1 — Sequence of 3C ligation products formed between latency control regions (129216F) and ORF50 promoter (69094R) or 3′ end of latency transcript (111485R). Sequencing chromatogram for 3C generated PCR products amplified with primers 129216F and 69094 (top panel) or with 129216F and 111484R (lower panel). (TIF) [file ppat.1002140.s001.tif]

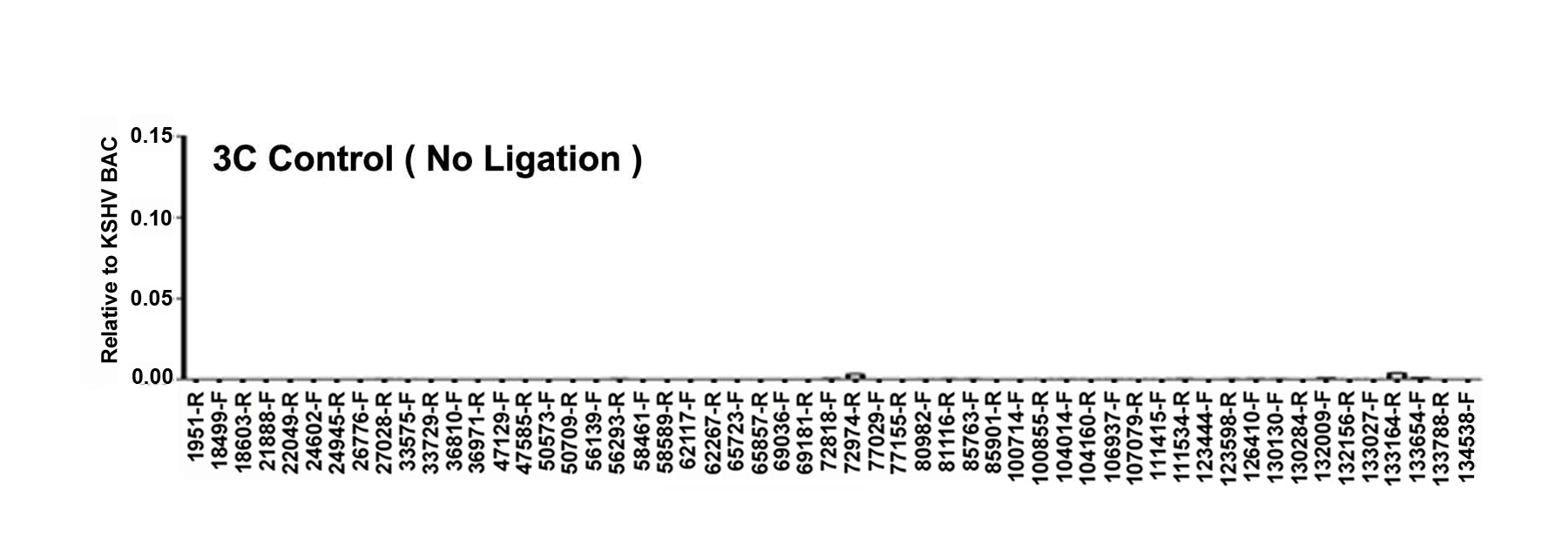

Supplement: Figure S2 — No Ligation Control for 3C. BCBL1 cells were treated essentially identically to cells treated in experiments shown in Fig. 1, but ligase was eliminated from the 3C protocol. These results show that all 3C signals are dependent on the ligation reaction. (TIF) [file ppat.1002140.s002.tif]

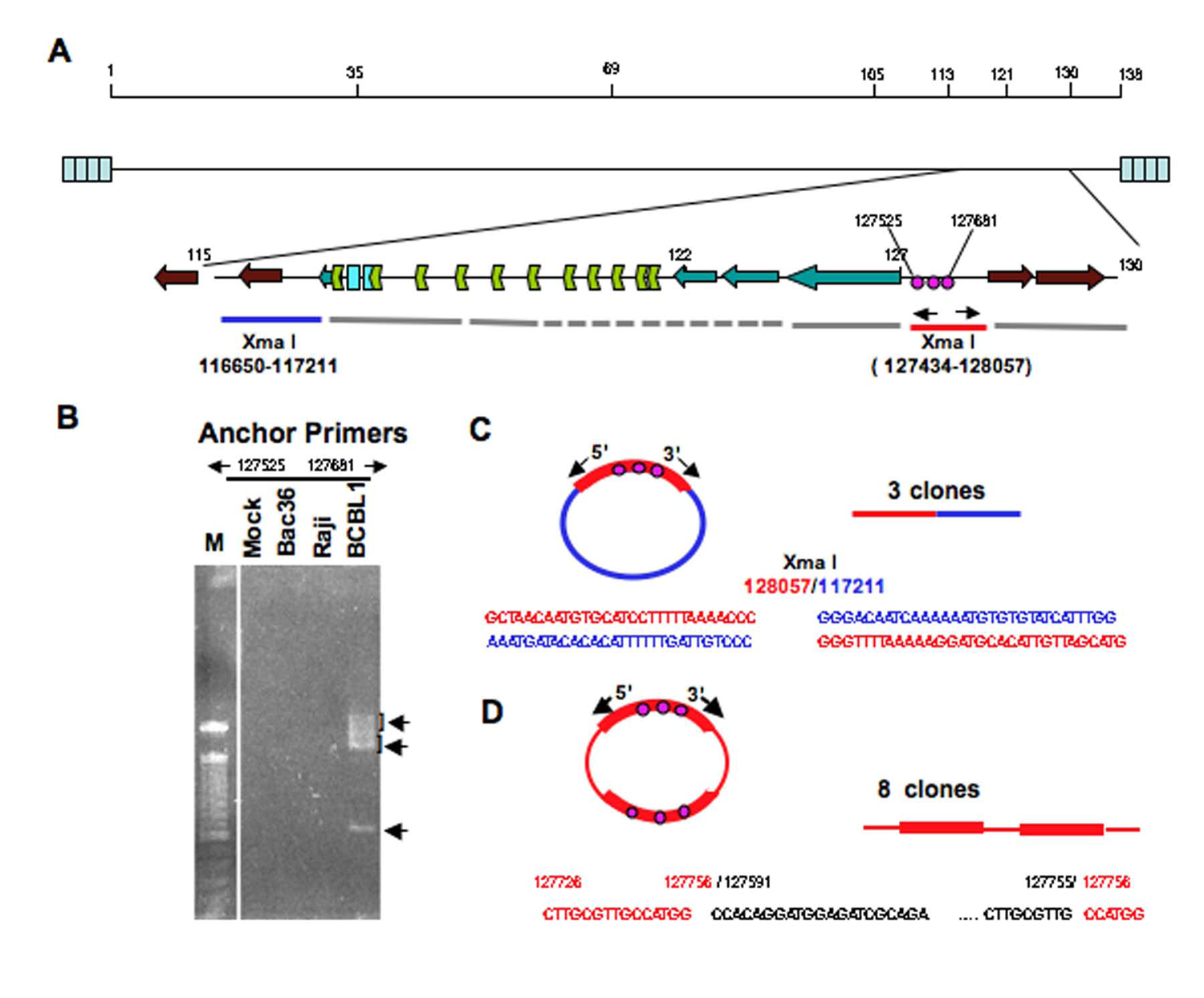

Supplement: Figure S3 — Circular Chromosome Conformation Capture (4C) of CTCF-cohesin site. A) Schematic of KSHV latency control region and XmaI restriction fragments. Anchor primers within 127434-128057 were used for inverse PCR and nested PCR amplification. B) Fragments amplified from 4C experiment with either mock (non-ligated DNA), purified Bac36 DNA, Raji cell, or BCBL1 cell DNA were fractionated by agarose gel electrophoresis. M is 1 kB DNA ladder. Arrows indicate fragments clones into TA-cloning vector. C) Schematic depiction of 3 clones containing CTCF-cohesin XmaI site fused to the K12 3′ end (Xma I site at 117211). D) Schematic depition of 8 clones showing a tandem duplication of the CTCF-cohesin binding site indicative of self-interactions. (TIF) [file ppat.1002140.s003.tif]

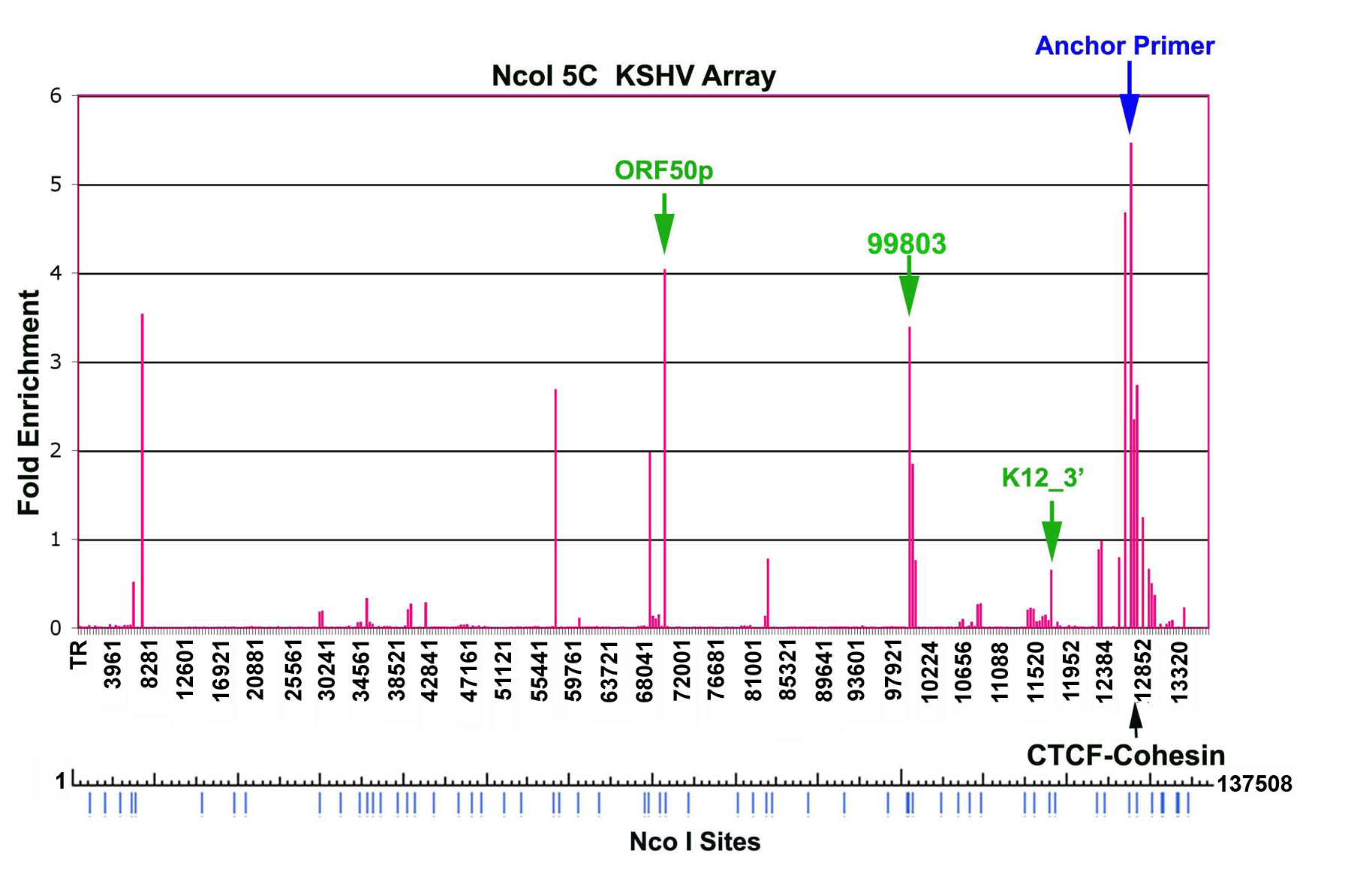

Supplement: Figure S4 — KSHV genome-wide PCR array analysis of chromatin conformation capture carbon copy (5C) products with anchor at CTCF-cohesin site. BCBL1 cells were processed for 3C analysis using NcoI to fragment the genome and inverse PCR primers anchored around the CTCF-cohesin binding site at 127,450. 3C amplified products were then quantified by real-time PCR using a KSHV genome-wide array. Peaks are indicated at the anchor site, K12 3′ end, Rta (ORF50) promoter, and position 99803. (TIF) [file ppat.1002140.s004.tif]

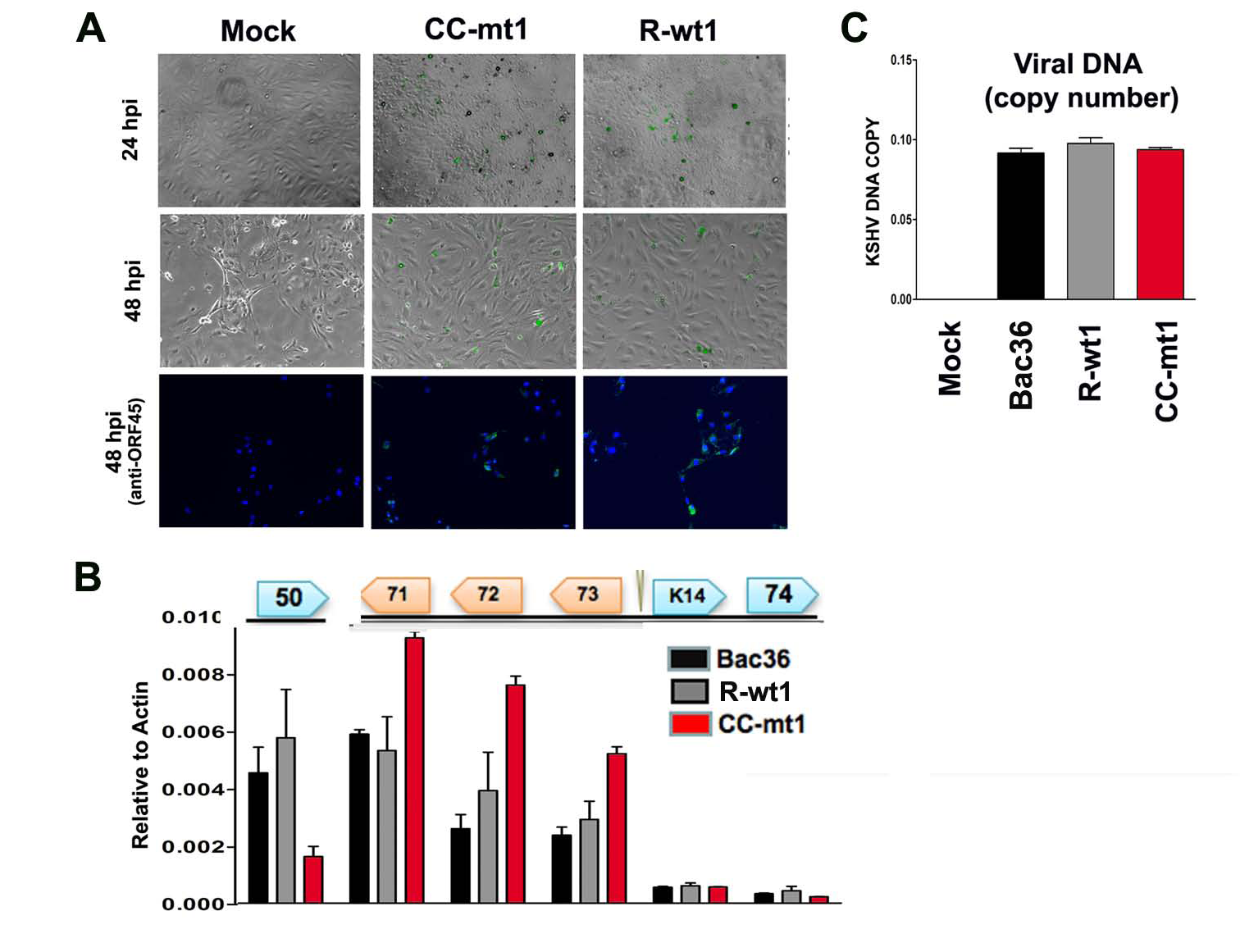

Supplement: Figure S5 — CTCF-cohesin site is required for control of latent and lytic transcription in primary infection of HUVEC cells. A) HUVEC cell infection with mock, CC-mt1, or R-wt1 bacmid derived virus assayed for GFP at 24 hr or 48 hrs, or for KSHV ORF45 at 48 hrs post infection. B) Intracellular viral DNA copy number in HUVEC infected cells at 48 hrs post infection virus from Bac36, R-wt1 or CC-mt1 genomes. C) RT-PCR of KSHV gene expression relative to cellular actin for HUVEC cells infected with Bac36 (black), R-wt1 (gray), or CC-mt1 (red). KSHV genes are indicated above each bar graph. (TIF) [file ppat.1002140.s005.tif]

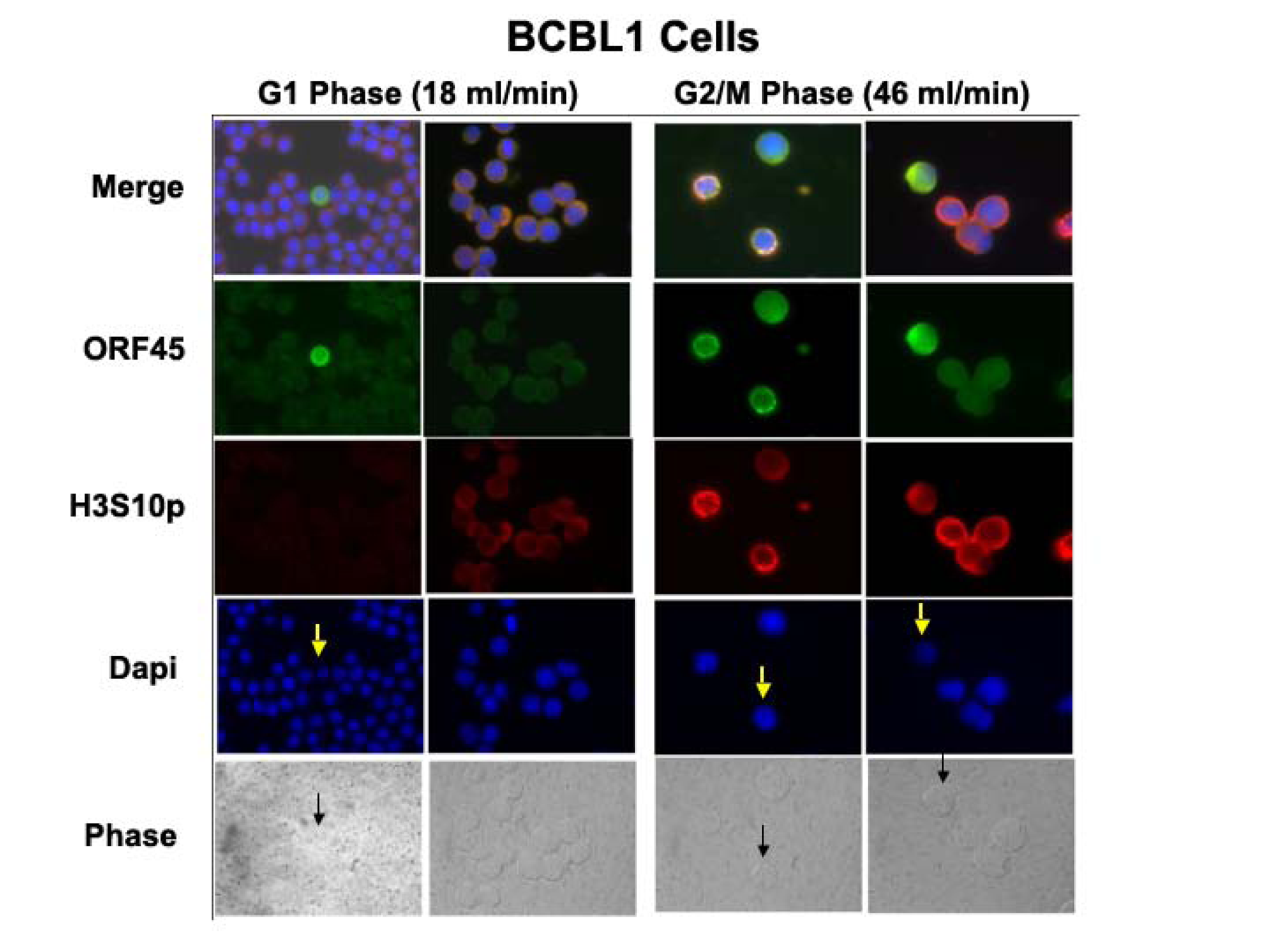

Supplement: Figure S6 — Increase in lytic gene expression in G2/M cells. BCBL1 cells were fractionated by centrifugal elutriation as described for Fig. 6. Cells from the G1 phase (18 ml/min) or G2/M phase (46 ml/min) were mounted by cytospin, fixed with paraformaldehyde, counterstained with DAPI, and then assayed for indirect immunofluorescence with antibodies to ORF45 (green), or histone H3 phosphoS10 (H3pS10) (red). These findings show that the majority of G2/M cells, as indicated by H3pS10 positive, are also positive for ORF45. This indicates that lytic cells are not a small subfraction of those cells elutriated during G2/M. (TIF) [file ppat.1002140.s006.tif]
